# Prenatal Maternal Anxiety in South Asia: A Rapid Best-Fit Framework Synthesis

**DOI:** 10.3389/fpsyt.2018.00467

**Published:** 2018-10-11

**Authors:** Katherine Stuart Bright, Jill M. Norris, Nicole L. Letourneau, Melanie King Rosario, Shahirose S. Premji

**Affiliations:** Faculty of Nursing, University of Calgary, Calgary, AB, Canada

**Keywords:** evidence synthesis, low- and middle-income countries, mental health, prenatal maternal anxiety, South Asia

## Abstract

**Background:** Most research efforts toward prenatal maternal anxiety has been situated in high-income countries. In contrast, research from low- and middle-income countries has focused on maternal depression and prenatal maternal anxiety in low- and middle-income countries remains poorly understood.

**Objectives:** To examine whether dimensions and attributes of current maternal anxiety assessment tools appropriately capture South Asia women's experiences of perinatal distress during pregnancy.

**Design:** We conducted a rapid review with best fit framework synthesis, as we wished to map study findings to an *a priori* framework of dimensions measured by prenatal maternal anxiety tools.

**Data Sources:** We searched MEDLINE, PsycINFO, and CINAHL and gray literature in November 2016. Studies were included if published in English, used any study design, and focused on women's experiences of prenatal/antenatal anxiety in South Asia.

**Review Methods:** Study quality was assessed using the Effective Public Health Practice Project Quality Assessment Tool and Critical Appraisal Skills Programme Qualitative Checklist. Study findings were extracted to an *a priori* framework derived from pregnancy-related anxiety tools.

**Results:** From 4,177 citations, 9 studies with 19,251 women were included. Study findings mapped to the *a priori* framework apart from *body image*. A new theme, *gender inequality*, emerged from the studies and was overtly examined through gender disparity, gender preference of fetus, or domestic violence.

**Conclusions:** Gender inequality and societal acceptability of domestic violence in South Asian women contextualizes the experience of prenatal maternal anxiety. Pregnancy-related anxiety tools should include domains related to gender inequality to better understand their influence on pregnancy outcomes.

## Introduction

Most of the research efforts toward prenatal maternal anxiety has been situated in high-income countries. In low- and middle-income countries (LMIC), other mental health issues such as maternal depression have remained a key focus of research and healthcare (1). Because of this, prenatal maternal anxiety in LMIC is poorly understood (2). Contextual factors in LMICs must be taken into consideration, such as high mortality rates for mothers and infants (3), poor access to healthcare (4), cost of obtaining care (5), and poor quality of available resources (6). Indeed, it has been suggested that aspects of prenatal maternal anxiety that are particularly potent may not exist only during the pregnancy, but be magnified due to the existence of the pregnancy itself (7), particularly in LMICs. Moreover, as the tools developed to measure anxiety have been developed in high-income countries, they may not map well on to the experience of women in LMIC who have anxiety.

Anxiety is traditionally differentiated into two factors: state anxiety and trait anxiety. State anxiety is dependent on situational or contextual circumstances, while trait anxiety is based on an individual's tendency to experience anxiety or one's disposition (8). Prenatal maternal anxiety include trait, state, and pregnancy-specific anxiety (9). Trait anxiety is defined as the stable aspect of an individual's personality whereas state anxiety is the temporary sensation of tension or worry in response to a stressful situation (9). Prenatal maternal anxiety is a distinct and definable syndrome that refers to fears and worries about the pregnancy, delivery, health of the child, and ability to care for the child (10, 11). Prenatal maternal anxiety incorporates both dispositional characteristics and environmentally influenced states (11). For example, environmental characteristics, in particular vulnerabilities that may exist outside the pregnancy, may enhance the social, cultural, societal, and environmental conditions experienced during pregnancy that increase levels of prenatal maternal anxiety (11).

Although prenatal maternal anxiety is a relatively new concept in maternal and child health, it is thought to be among the most influential risk factors affecting both maternal and child outcomes (9, 11). The most commonly cited adverse effects associated with prenatal maternal anxiety are preterm birth and low birth weight (11–16), both of which have global consequences for infant mortality and morbidity (11). Preterm birth can lead to numerous physical and cognitive complications, including cerebral palsy, cognitive impairment, and respiratory ailments (17–19).

Other maternal adverse outcomes related to prenatal maternal anxiety include effects on length of labor (20) Prenatal maternal anxiety can have a direct effect on the fetus including altering fetal heart rate (21), and increase the risk for postnatal depression (22, 23). The relationship between mother and infant may be altered by maternal anxiety, resulting in lack of engagement, and interaction with the infant (24). The impact of the lack of engagement between mother and infant may result in neurological and emotional development alterations in the infant and affect their ability to adapt, regulate, and moderate behavior (25–30). Additional studies have reported an association between the physiological effects of anxiety in pregnancy and child neurodevelopment, including developmental delays (31, 32), negative childhood emotionality (33), variances in attention deficit hyperactivity disorder, and anxious symptoms at various ages during childhood (34–36).

Prenatal mental health concerns are particularly prevalent in LMIC, where access to mental health services is scarce (1, 37), inequitably distributed between regions (38), and contributes to preterm births (9, 11). There are twelve countries that account for more than 60% of these preterm births, eight of which are LMICs (39). In many LMICs, they are reducing the incidence of preterm deliveries except for Pakistan, India, and Bangladesh (39, 40). These three nations are a part of the eight, Bangladesh, Afghanistan, Nepal, India, Pakistan, Maldives, Bhutan, and Sri Lanka, that are comprised to form South Asia (41). Despite regional, religious, spiritual, and language differences, South Asia has a shared cultural identity (42).

There are several self-reporting scales to detect symptoms of prenatal maternal anxiety, which were developed in English-speaking high-income countries (10, 43–46) and translated into other languages for the use in LMICs. Although these anxiety measures may be valid for screening in high-income countries, they have not been validated in LMIC. As a result, there is debate about whether these anxiety instruments have the same sensitivity and specificity in different cultural settings (47). The concerns focus on whether these psychological constructs are equivalent dimensions between cultures, populations, and groups and the different response patterns between settings. Given these concerns—and the importance of improving our understanding of prenatal maternal anxiety in South Asia—we wanted to clarify whether current prenatal maternal anxiety measurement tools capture the dimensions of anxiety during pregnancy that are experienced by women in South Asia, and if there are features of anxiety during pregnancy that are unique to women in South Asia.

## Methods

### Objective

The objectives of this rapid review were to examine how dimensions and attributes of current maternal anxiety assessment tools appropriately capture South Asia women's experiences of perinatal distress during pregnancy.

### Design

Our review team included content experts and a knowledge synthesis methodologist. We conducted a rapid review (48), in order to conduct a streamlined and time-limited evidence review, with a best-fit framework synthesis (49, 50). Best-fit framework synthesis was appropriate for our objectives as we wished to map study findings to an *a priori* framework of dimensions measured by prenatal maternal anxiety tools. While there are currently no theories of prenatal maternal anxiety specifically, a recent concept analysis proposed 11 dimensions measured by prenatal maternal anxiety tools (51). We used the 11 dimensions to construct an initial or “best fit” framework (Table 1), as we anticipated that our synthesis would generate context-specific dimensions and extend beyond the initial framework. ENhancing Transparency in REporting the synthesis of Qualitative research (ENTREQ) statement was used for reporting (52) see Supplementary Datasheet 1.

**Table 1:** *A priori* framework (51).

**Dimension**	**Definition**
Fetal health	Healthy development of her fetus
Loss of fetus	Miscarriage, stillbirth, or preterm birth
Childbirth	Pain during delivery and complication in delivery
Mother's well-being	Physical and mental health concerns during pregnancy and postpartum period
Body image	Weight gain and physical appearance in pregnancy
Parenting and care for child	Capacity bond with baby and competency in her mothering skills/ensuring healthy and safety of baby
Healthcare related	Access to antenatal care and ability to have autonomy in healthcare decision-making
Financial	Economic security including employment, housing, and ability to meet the cost of raising a child
Family and social support	Insufficient partner and/or immediate family/in-law support during pregnancy/postpartum, loss of freedom, and concerns around the acceptance of the gender of the baby
Pregnancy general	Non-specific aspects of pregnancy
Confidence and control	Certainty about and power over labor or the outcome of the pregnancy

### Search methods

We created a comprehensive search strategy in consultation with an information scientist, with searches of MEDLINE (Ovid), PsycINFO, and CINAHL. The searches were conducted in November 2016. The MEDLINE search strategy is detailed in the Supplementary Datasheet 2. Search terms and subject headings for prenatal maternal anxiety were adapted for each database and combined with the countries identified in the Cochrane Effective Practice and Organization of Care Group - Norwegian Satellite (53) LMIC search filter, without limits. Gray literature searches were conducted using forward citation searches in Google Scholar. Reference lists of the included studies were reviewed to identify additional relevant literature.

### Eligibility criteria

As part of a larger review, we initially included English-language studies of any design that reported on women's experiences of prenatal or antenatal anxiety in LMICs. At the full-text screening stage, we narrowed the inclusion criteria to include only studies based in South Asia. Upon completion of the full-text review, we discovered that our initial inclusion criteria (all LMICs) would be unacceptable because of significant differences in prenatal maternal anxiety antecedents between countries (i.e., social, cultural, religion, and AIDS). By focusing on South Asia, we could better examine cultural, religious, health concerns, and social factors contributing to anxiety in pregnancy. Studies that described postpartum anxiety without extractable data for prenatal or antenatal anxiety were excluded.

### Study selection

Results of all searches were saved in each database and imported into EndNote, where duplicates were removed. One reviewer independently screened titles and abstracts using a standardized screening tool. Full texts of potentially relevant studies were obtained and screened for eligibility.

### Quality assessment

Studies were not excluded based on quality. We used the Effective Public Health Practice Project Quality Assessment Tool [EPHPP; (54)], which has six domains assessed as *strong, moderate, weak*, or *not applicable*: selection bias, study design, confounders, blinding, data collection methods, and withdrawals/drop outs. We also used the Critical Appraisal Skills Programme Qualitative Checklist [CASP (55)]. For qualitative studies, each of the 10 domains were assessed as *no, yes*, or *can't tell*: aims, methodology, design, recruitment, data collection, researcher-participant relationship, ethical issues, data analysis, finding, and value. For mixed methods studies, we used both tools. One reviewer appraised the studies for methodological quality. A second independent reviewer verified the accuracy of the quality assessment for each study, and disagreements were resolved through discussion.

### Data extraction and synthesis

One reviewer extracted data in Excel from the included studies including the following data items: study, year, country, purpose, study design, participants (eligibility, response rates, characteristics), methods (data collection and analysis). The results of studies were extracted into the *a priori* framework. Where data did not fit into the framework, data was synthesized using thematic analysis and additional themes were added to the framework. A second reviewer verified all extracted data to ensure accuracy, and any disagreements were resolved through discussion.

## Results

### Study selection and characteristics

**Figure 1:**
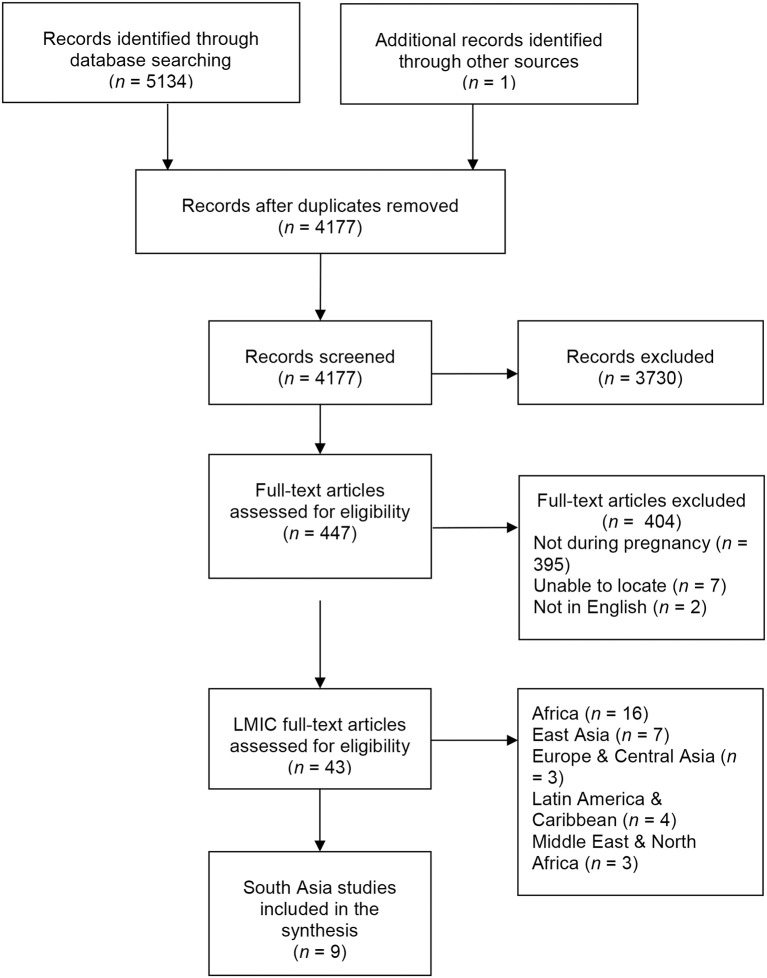
PRISMA flow diagram.

Figure 1 illustrates the flow of studies through the review process. From 5,134 citations, we screened 4,177 titles and abstracts. A total of 447 full-text articles were screened for eligibility, from which 43 studies from LMICs were eligible. Overall, eight studies (nine articles) from South Asia were included in the synthesis (see Table 2 for study characteristics). Studies were published between 2006 and 2015, and this synthesis includes 19,251 women across Pakistan (*n* = 6), India (*n* = 1), Bangladesh (*n* = 1), and Nepal (*n* = 1). Participants exhibited varied rates of literacy or schooling (illiterate/no formal schooling, 17–66%) with mean ages between 23.8 and 27.9 years. Study sample sizes ranged from 79 to 9,078. Seven studies used a cross-sectional design and two studies were tool-development or validation studies. While Kazi et al. (59) included qualitative interviews during the development of a new stress in pregnancy scale, they did not present any qualitative data beyond potential items generated.

**Table 2:** Included studies.

**Study**	**Region**	**Purpose**	**Design**	**Sample**	**Anxiety tool**
Karmaliani et al. (56)	Hyderabad, Pakistan	Explore psychometric properties of two scales to identify which scale is the most appropriate measure of depression and anxiety	Cross-sectional	997 pregnant women, 20–26 week's gestation	Aga Khan University Anxiety and Depression Scale, How I Feel scales
Karmaliani et al. (57)	Hyderabad, Pakistan	Assess the diagnostic validity of two scales for use in assessing depression and anxiety among pregnant women	Psychometric validation	200 pregnant women, week's gestation not known	Aga Khan University Anxiety and Depression Scale, How I Feel scales, DSM IV interview
Karmaliani et al. (58)	Hyderabad, Pakistan	Estimate the prevalence of and identify precursors for antenatal psychological distress among women	Cross-sectional	1,369 pregnant women, 20–26 week's gestation	Aga Khan University Anxiety and Depression Scale
Kazi et al. (59)	Karachi, Pakistan	Develop an appropriate and comprehensive scale based on stressors for measuring stress among pregnant women	Tool development and validation with interviews	Pregnant women: 79 Phase 1, 342 Phase 2, week's gestation not known Experts: 25 Phase 1	Interviews, A–Z scale
Nasreen et al. (60)	Rural Bangladesh	Examine and identify the prevalence of potential contributors to antepartum anxiety and depressive symptoms among women	Cross-sectional	720 pregnant women in third trimester, week's gestation not known	State-Trait Anxiety Inventory
Prost et al. (61)	Rural tribal areas, Eastern India	Identify socio-economic, gender, and health related predictors of maternal psychological distress among recently delivered mothers	Cross-sectional	5,801 postpartum women	Kessler 10
Ali et al. (62)	Karachi, Pakistan	Estimate the frequency and associated risk factors for depression and anxiety in pregnant women	Cross-sectional	165 pregnant women, week's gestation not known	Hospital Anxiety and Depression Scale
Clarke et al. (63)	Dhanusha district of Terai region, Nepal	Identify factors associated with psychological distress among mothers.	Cross-sectional	9,078 postpartum women	General Health Questionnaire 12
Waqas et al. (64)	Lahore, Pakistan	Investigate factors associated with antenatal depression and anxiety, including gender discrimination and preference for sons	Cross-sectional	500 pregnant women with lower income, week's gestation not known	Hospital Anxiety and Depression Scale

### Quality assessment

Overall, the studies were generally moderate in quality. A summary of the rating of individual items of the EPHPP tool (54) is reported in Table 3.

**Table 3:** Quality appraisal.

**Study**	**Selection bias**	**Study design**	**Confounders**	**Blinding**	**Data collection methods**	**Withdrawal, drop outs**
Karmaliani et al. (56)	M	M	N/A	N/A	M	M
Karmaliani et al. (57)	M	M	S	N/A	S	W
Karmaliani et al. (58)	M	M	N/A	N/A	M	M
Kazi et al. (59)	S	M	M	N/A	S	S
Nasreen et al. (60)	M	M	S	N/A	S	W
Ali et al. (62)	W	M	S	N/A	M	W
Prost et al. (61)	W	W	S	N/A	M	M
Clarke et al. (63)	S	S	S	N/A	S	W
Waqas et al. (64)	M	M	M	N/A	S	W

*S, strong; M, moderate; W, weak; N/A, not applicable*.

### Findings

Data from the included studies supported the *a priori* framework except for one concept that was not present: body image. None of the studies assessed or discussed body image. One new higher-order theme, *gender inequality*, was added to the framework. Gender inequality was overtly examined through the gender disparity, gender preference of fetus, and/or domestic violence. The examination of the cultural context of prenatal maternal anxiety revealed many areas within the South Asian society where gender inequality exists covertly throughout included maternal well-being and access to healthcare, opportunities for education and employment outside the home, maternal decision-making, control, and confidence. Table 4 summarizes the new framework informed by this review, with examples from the included studies.

**Table 4:** New framework of prenatal maternal anxiety for South Asian context.

**Dimension**	
Gender inequality	•Preference for sons (64)
	•Maternal decision-making control over daily household expenditures and health-seeking behaviors for herself (61)
	•Infant gender preference for a son (63)
	•Household decision-making (62)
	•Female employment outside of the home (64)
Domestic violence	•Sexual, physical and verbal abuse by any family member (62)
	•Physical, sexual, or verbal abuse within 6 months of current pregnancy (60)
	•Verbal abuse by husband (59)
	•Harassment (64)
Fetal health	•Appearance of the unborn baby (59)
Loss of fetus	•Previous losses, neonatal death, and childhood death (60)
	•Stillbirth and neonatal death (61)
	•Previous miscarriage/stillbirth/infant death (63)
	•Previous miscarriages (64)
Childbirth	•Previous method of delivery and adverse outcomes of pregnancies (61)
	•Adverse outcomes in previous pregnancies (62)
Mother's well-being	•Health problems in pregnancy, delivery, and postpartum (61)
	•Increased maternal age (61)
	•Maternal age when she got married (63)
Parenting and care for child	•Higher parity (63)
	•Looking after the children and concerns about children's illness (59)
Healthcare related	•Antenatal care received (63)
	•Access to quality reproductive health services with integrated mental health services (61)
	•Abortion care including post-abortion surgical care, family planning methods, and care provided by untrained healthcare professionals called “dai” (64)
	•Inaccessibility of healthcare and seeking help for reducing their worries (62)
Financial	•Concerns about access to husband's money, husband's unstable job, husband's unemployment, owing money, insufficient money for buying the house, and increase in the process of everyday foods (59)
	•Concerns about food security (63)
	•Total land owned within the household economic status (60)
	•Husband's lack of unemployment and low property index (58)
Family and social support	•Concerns about missing her own parents, less socialization due to pregnancy, and gaining supremacy among in-laws (59)
	•Not having her own family support in the postpartum period (63)
	•Poor relationships with husbands and poor practical support in pregnancy (60)
	•Lack of social support (64)
Pregnancy general	•Concerns around feeling unwell during pregnancy, delay in household work due to pregnancy, and an unwanted pregnancy (59)
	•Willingness of pregnancy (62)
	•Time of concern regarding gender of the infant and that women who had not already given birth to a son (63)
	•Unwanted pregnancy (61)
	•Unplanned pregnancy (60)
	•Unplanned pregnancy (64)
Decision-making, control and confidence	•Concerns about not having freedom to make decisions that explored both control and decision-making for women (59)
	•Husband and in-laws were the main decision-makers for daily household expenditures (61)
	•Decision-making around reproductive health and family planning method use (62)
	•Experiencing difficulties in thinking clearly, feeling hopeless, and feeling helpless (58)
	•Having nothing to be proud of, having little control over thing that happen to her, and feeling helpless in dealing with problems of life (56)

### Gender inequality

The relationship between gender inequality and level of prenatal maternal anxiety was prevalent in five of the nine articles. Four articles discussed gender inequality in terms of gender preference of the baby, gender inequality through domestic violence, restricting diet in the postpartum period, and decision-making about household task and personal healthcare. Having daughters was significantly associated with increased levels of anxiety in pregnancy (64). For women who have already given birth to a daughter the family and societal pressure to have a son is even greater, significantly contributing to prenatal maternal anxiety (*p* = 0.01) (64). Prost et al. (61) described gender-related factors as including maternal decision-making power around daily household expenditure and health-seeking for herself in case of illness, and food restrictions in the postpartum period. They found that there was no significant association between gender of the infant and maternal anxiety in pregnancy. Gender-based factors were assessed in the study by Clarke et al. (63), which determined that pregnancy was a time of concern regarding gender of the infant, as well as restricting diet during the postpartum period. Women who had not already given birth to a son had significantly higher risk of psychological distress [β 1.25 (95% CI 1.08–1.44); *p* = 0.003]. While there was no significant association between domestic violence and maternal anxiety and/or depression in Ali et al. (62), there was a significant association with psychological distress status and household decision-making (*p* = 0.018). Interestingly, six articles discussed the consequences of female work and maternal anxiety. Waqas et al. (64) reported that pregnant women, working outside the home, were more anxious than housewives (*p* < 0.001). Factors contributing to women working outside the home and their higher levels of anxiety include the deteriorating socio-economic situation in Pakistan, economic needs of their household, negative attitudes, and stigma toward women who had to secure employment.

#### Gender inequity and domestic violence

Within the South Asian literature, domestic violence takes the form of sexual, physical, and verbal abuse by any family. A total of 79.2% of women (*n* = 132) were forced to have sex with their husbands, 33.8% (*n* = 244) of women experienced multiple acts of physical violence, and 18.1% (*n* = 130) had experienced physical violence in their pregnancies (62). Nasreen et al. (60) reported that during pregnancy 15% (*n* = 208) of the overall sample of women had experienced physical and/or sexual abuse and 30% (*n* = 408) had experienced verbal abuse only within six months of their current pregnancy. Interestingly, Kazi et al. (59) included one item, *concerns about verbal abuse by husband*, in their scale development article and did not explore the concerns of physical and/or sexual abuse. Waqas et al. (64) reported the experience of harassment at a rate of 6.6% (*n* = 3; *p* < 0.01) but did not define harassment as being domestic violence.

### Fetal health

One study included the concept of fetal health. After interviewing 79 women to generate potential survey items, Kazi et al. (59) developed and tested a tool to assess stressors during pregnancy within the South Asian context. The tool included an item on the concern about the appearance of the unborn baby. Their study did not diagnose or label women with stress or anxiety, rather it provided a total score suggestive of the level of stress.

### Loss of fetus/previous stillbirth or neonatal death

Four studies explored fetal/infant loss in the context of pregnancy-related anxiety (60, 61, 63, 64). Across these studies, between 4 and 25% of study participants had experienced fetal/infant loss. In the Prost et al. (61) study from rural India, 7.9% of women reported stillbirth and neonatal death; from these women, 39.2% also experienced distress during their current pregnancy. Loss of an infant significantly increased the risk for distress [AOR 7.06 (95% CI 5.51–9.04); *p* < 0.005]. Previous miscarriages were reported in 8.8% of the Pakistani women in one cross-sectional study (64). Anxiety exhibited a small correlation with history of miscarriages (*r* = 0.10, *p* = 0.05). Obstetrical indicators such as previous losses, neonatal death, and childhood death were assessed in a cross-sectional study from Bangladesh (60). Of the 342 mothers, 25% experienced the loss of one to three children from miscarriage and stillbirth. History of child death was not predictive of general anxiety symptoms (*p* = 0.302). Similarly, a previous miscarriage, stillbirth, or infant death did not significantly increase the risk of distress in women from rural Nepal [β 1.18 (95% CI 0.90–1.54); *p* = 0.223] (63).

### Childbirth

Both Prost et al. (61) and Ali et al. (62) mentioned previous delivery methods and adverse outcomes of pregnancies impacting distress in current pregnancy. Prost et al. (61) reported that 1.7% of women had a history of cesarean section, which significantly increased the risk of stress [AOR 1.95 (95% CI 1.12–3.40); *p* = 0.018]. Ali et al. (62) reported that 31.1% of women in their study had an adverse pregnancy outcome, which also substantially increased their risk for anxiety [AOR 3.25 (95% CI 1.09–9.70); *p* = 0.013].

### Mother's well-being

Only one study, Prost et al. (61), reported maternal well-being in terms of health problems in pregnancy (46.3%), delivery (35.1%), and postpartum (30.5%) for 5,801 women in rural India. The risk of distress significantly increased health problems during the pregnancy [AOR 1.98 (95% CI 1.62–2.41); *p* < 0.005], during delivery [AOR 1.63 (95% CI 1.34–1.99); *p* < 0.005], and during the postpartum [AOR 1.95 (95% CI 1.12–3.40); *p* < 0.005], as well as with increased maternal age [AOR 1.44 (95% CI 1.03–2.07); *p* = 0.040]. Comparatively, Clarke et al. (63) did not find an association between maternal age at pregnancy and distress (*p* = 0.551) but noted that women who married between 16 and 17 years of age had a higher risk of experiencing anxiety during pregnancy [β 1.18 (95% CI 1.01–1.37); *p* = 0.034].

### Parenting and care for the child

Two studies, Kazi et al. (59) and Clarke et al. (63), explored prenatal distress coupled with parenting and care for the children. In the Kazi et al. (59) tool development study, interviews generated two parenting-related items, *concerns about children's illness*, and *concerns about looking after the children*. A related finding from Clarke et al. (63) demonstrated that women who had five or more children at home (9.1%) were at risk for distress [β 1.34 (95% CI 0.09–1.66); *p* = 0.006].

### Healthcare related

Three studies examined healthcare in relationship to prenatal maternal anxiety. Clarke et al.(63) reported that there was a significant difference in level of prenatal psychological distress between individuals who received no antenatal care [OR 1.30 (95% CI 1.15–1.461); *p* < 0.001] and some antenatal care [OR 1.33 (95% CI 1.09–1.61); *p* < 0.001] and those who received satisfactory care. Prost et al. (61) described healthcare as access to quality reproductive health services with integrated mental health services and, although no statistical analysis was performed on this item, there was discussion regarding the testing of strategies to integrate mental health treatments into primary care services in underserved areas of India. Healthcare was also discussed by Waqas et al. (64), albeit in relation to abortion care, including post-abortion surgical care, family planning methods, and care provided by untrained healthcare professionals called “dai.” Waqas et al. (64) also discussed rural and urban discrepancies in the provision of healthcare in Pakistan, and recommended to develop programs in rural communities that are aimed at increasing health facilities, education, and resources for reducing psychological distress in pregnant women. Lastly, Kazi et al. (59) included in their pregnancy stress scale framework development concerns about inaccessibility of healthcare. Only one study discussed whether women sought help for their mental health concerns and whether there was any impact on maternal anxiety in current pregnancy. In the Ali et al. (62) study, 20.8% (*n* = 30) of women did seek help for reducing their worries yet continued to experience symptoms of anxiety.

### Financial conditions of poverty

Several articles in this review discussed prenatal maternal anxiety in relationship to financial concerns and conditions of poverty. In Kazi et al. (59), 20% of their framework items involved socio-economic/financial concerns and included items such as concerns about access to husband's money, husband's unstable job, husband's unemployment, owing money, insufficient money for buying the house, and increase in the process of everyday foods. Clarke et al. (63) measured socio-economic/financial concerns in terms of food security. When food was moderately or severely insecure, these pregnant women from Nepal reported psychological distress that was statistically significant [β: 1.36 (95% CI 1.04–1.77); *p* = 0.023 and β: 2.21 (95% CI 1.43–3.40); *p* < 0.001, respectively]. Nasreen et al. (60) assessed socio-economic/financial aspects of pregnancy-related anxiety including total land owned within the household (economic status) in their study of women from Bangladesh. The model did not indicate a direct association between poor economic status and general anxiety symptoms. In the study of women from Pakistan, Karmaliani et al. (58) noted significant relationships between psychological distress in pregnancy and two financial items including husband's lack of employment [OR 2.10 (95% CI 1.07–4.12); *p* = 0.032] and low property index, a measure of household ownership (i.e., home, land, vehicle, television, refrigerator) [OR 1.44 (95% CI 1.04–1.99); *p* = 0.027].

### Family and social support

Kazi et al. (59) included in their stress items in pregnant women concerns about missing her own parents, less socialization due to pregnancy, and gaining supremacy among in-laws. These three items exploring family and social support had high item total correlation, 0.30 and higher. Clarke et al. (63) reported that not having their own family support in the postpartum period contributed to increased levels of psychological distress [β 1.14 (95% CI 1.00–1.29); *p* = 0.044]. Nasreen et al. (60) reported statistically significant increase in symptoms of anxiety and when women experienced poor relationships with husbands and poor practical support in pregnancy (*p* < 0.001 and *p* = 0.021, respectively). The significant impact that the lack of social support has on increasing anxiety in pregnant women (*p* < 0.001) was also determined in Waqas et al. (64).

### Pregnancy in general

Kazi et al. (59) included in their development of the stress scale in pregnancy items around pregnancy in general including concerns around feeling unwell during pregnancy, delay in household work due to pregnancy, and an unwanted pregnancy. Nasreen et al. (60) noted that there was no significant change in anxiety level for women in Bangladesh if the pregnancy was unplanned while Waqas et al. (64) noted that women in Pakistan experienced statistically significant higher levels of anxiety when the pregnancy was not planned (*p* < 0.001).

### Control and confidence

The scale development article by Kazi et al. (59) included the stressor item *concerns about not having freedom to make decisions* that explored both control and decision-making for women in Pakistan. Prost et al. (61) reported that husband and in-laws were the main decision-makers for daily household expenditures increased the risk for maternal anxiety [OR 0.55 (95% CI 0.37–0.80); *p* = 0.002]. Ali et al. (62) examined decision-making around reproductive health and whether women could decide about family planning method use. They determined that 16.7% (*n* = 13) of women who were anxious were also not able to make the decision about family planning method, while 37.2% (*n* = 14) of women who were anxious and depressed were not able to be decision-makers around family planning methods. With items from the Aga Khan University Anxiety and Depression Scale (AKUADS) in Karmaliani et al. (58); 8% of pregnant women reported always/often experiencing difficulties in thinking clearly, 10% feeling hopeless always/often, and 10% feeling helpless always/often.

## Discussion

This paper reported the experiences of prenatal maternal anxiety in South Asia through a rapid review, with the goal of informing cultural adaptations to prenatal maternal anxiety scales. The aspects of anxiety-producing thoughts for pregnant women in middle-high countries and South Asia that map on to current pregnancy-related anxiety tools include: fetal health, loss of fetus, childbirth, parenting/caring for the child, healthcare related, financial issues, social support, pregnancy in general, and control/confidence. Although many of the dimensions of prenatal maternal anxiety in South Asia may map on to current tools, it is the intensity of these stressors that distinguish them from the experiences of women in middle-high income countries. For example, in middle-high income countries, pregnant women with anxiety-related to control/confidence may be inclined to choose a mode of delivery that provides them with a greater sense of control (51). Whereas, in the South Asia context control/confidence is related to women's inability to be decision-makers in any aspect of their own health as their husbands and in-laws are in charge of their reproductive health and family planning (59). We recommend that decision-making, control, and confidence, domestic violence, gender disparity/inequality between males and female, and gender preference of the fetus be considered in future prenatal maternal anxiety tools for use in South Asia. These added dimensions will assist in developing a tool that more accurately captures the sociocultural experience of prenatal maternal anxiety for women living in South Asia. However, without qualitative studies, we were not able to attain the rich descriptions of prenatal maternal anxiety from the perspectives of women that would significantly further our theoretical understanding of the concept in South Asia.

Within South Asia, gender inequalities appear to be a significant and overarching theme of the shared cultural understanding of prenatal maternal anxiety. The preference for sons over daughters is evident in all areas, from access to healthcare all the way to opportunities for advanced education (64). Gender constraints in the South Asian society are present throughout the varies castes and tribes (65). On a community level, gender division is a result of the prevailing societal and cultural norms of how males and females are perceived (66). These gender constraints occur on macro, meso, and micro levels. At the macro level, gender discrimination is based on the self-sustaining and deep traditions entrenched in culture and the political economy (67). On the meso level, gender constraints are contextualized at different individual, group, and organizational levels including the rules applied to and resources available to diverse individuals (68). Lastly, on the micro level is the context of individual identity, priorities, and relational perspectives including considering the impact that is fostered through individual interactions and relationships (67).

Domestic violence is the most prevalent form of gender-based violence worldwide (69, 70). Intimate partner violence is a global health issue with significant mental health and human rights consequences to women (71). Unfortunately, domestic violence is a common problem during pregnancy and is associated with increased risk of miscarriage, preterm delivery, and low birth weight babies (69). The occurrence of domestic violence is vastly uneven between middle-high and low-income countries (72, 73). Prevalence rates of domestic violence over the past 12 months are reported to be < 4% in high-income countries compared to ~40% in low-income countries (72). It has been reported that domestic violence arises from interactions among personal, situational, and sociocultural factors (74). Domestic violence is more likely to occur in geographical regions where there it socially and culturally acceptable for males to exert authority over female behaviors (72, 73). Domestic violence in South Asia is closely related to marriage-related norms and practices that reinforce women's lack of power (71). These male normative-related behaviors are associated with current laws and practices which impede women and justify wife beating (72). In South Asia, there are additional barriers to disclosing intimate partner violence which include cultural expectations of not disclosing family problems that could bring shame to a family's reputation and honor (75). There is a power imbalance within the marital relationship where gender roles clearly articulate male authority (69, 71). As a shift in power occurs between husband and wife, the result is often a husband's sense of control being undermined and this can lead to intimate partner violence (69). Factors contributing to a shift in power include women's economic contribution and level of education. The social and economic environment in South Asia has undergone rapid change and out of economic necessity, women are being exposed to new opportunities, changing norms, and are deviating from their traditional gender roles (70, 71, 74). There are normative perceptions on domestic violence in South Asia where husbands report a right over their wives and there is public tolerance for spousal abuse (70). Unfortunately, the inter-generational cycling of domestic violence, beaten as a child and witnessing abuse of the mother, perpetuates the view of violence as a normal behavior in conflict situations within the home (69, 70).

From a higher-income country perspective, domestic violence and gender inequality are viewed as breaches of human rights with serious public-health consequences that should be addressed in national and global health policies and programs (76). Over the last 20 years, domestic violence research has focused on interventions, supportive services for victims, and increasing effectiveness of the justice system in the context of high-income countries (73). In lower-income countries, domestic violence research has focused on violence prevention programs (73). In their systematic review of structural interventions for domestic violence in LMIC, Bourey et al. (77) suggested that prevention programs need to address social and economic risk, controlling behaviors, improved economic well-being, enhanced relationship quality, motivate help-seeking behaviors, reduce social acceptability of domestic violence, and create more equitable gender norms. Unfortunately, in LMICs, there is reluctance in supporting government involvement in resolving domestic violence. This may reflect a lack of belief in the effectiveness of government run program, or the perception that domestic violence is a private problem (70, 78). Thus, an effective solution to the problem of domestic violence would require involvement of community-based non-government organizations (70).

## Strengths and limitations

This rapid review included a systematic search with attempts to find gray literature, and we undertook a quality assessment of the included studies. Consistent with rapid review methods (79), we streamlined our process by not registering the protocol, limiting our review to English-language publications, and only having one reviewer screen the literature; these steps introduce potential bias to our findings. However, there is evidence that the conclusions produced by rapid reviews and systematic reviews are similar (80). One key limitation of the review was the lack of qualitative studies to synthesize, which hindered our in-depth understanding of the complex experience of pregnancy-related anxiety and our ability to provide comprehensive recommendations. Moreover, we purposefully limited this review to South Asian women, English-language studies, and did not include other LMICs, which may limit the generalizability of our findings.

## Conclusion

The results from this review indicate that gender inequality in South Asian women contextualizes the experience of prenatal maternal anxiety. With rapid change in the social and economic environment in South Asia, women have been exposed to new opportunities including economic contributions and level of education thus challenging their traditional gender roles and potentially contributing to intimate partner/domestic violence. The societal acceptability of domestic violence further contributes to this systemic gender inequality of women. It is suggested that non-government community-based programs be aimed at changing public discourse, practices, and norms for gender inequality and domestic violence. These intervention programs must be aimed at addressing social and economic risk, controlling behaviors, improving economic well-being, enhancing relationship quality, and motivating help-seeking behaviors. These programs may reduce social acceptability of domestic violence while simultaneously creating more equitable gender norms.

## Ethics statement

This review was exempt from ethical review.

## Authors contributions

KB, MK, NL, and SP conceived the idea for this review. KB, SP, and JN contributed to the methodology. KB conducted the search, data extraction, appraisal, and synthesis. JN verified the data extraction, appraisal, and synthesis. All authors prepared the initial draft and approved of the final draft.

### Conflict of interest statement

The authors declare that the research was conducted in the absence of any commercial or financial relationships that could be construed as a potential conflict of interest.
