# Supplementary material for: Prenatal Maternal Anxiety in South Asia: A Rapid Best-Fit Framework Synthesis
Source: Front Psychiatry. 2018 Oct 11;9:467. doi: 10.3389/fpsyt.2018.00467 (PMC6193096; doi:10.3389/fpsyt.2018.00467)
Supplement: Supplementary file 2 [file Data_Sheet_2.PDF]

Appendix

MEDLINE (Ovid) Search Strategy

1. (Afghanistan or Albania or Algeria or American Samoa or Angola or Armenia or Azerbaijan or Bangladesh or Belarus or Byelarus or Belorussia or Belize or Benin or Bhutan or Bolivia or Bosnia or Botswana or Brazil or Bulgaria or Burma or Burkina Faso or Burundi).mp.
2. (Cabo Verde or Cape verde or Cambodia or Cameroon or Central African Republic or Chad or China or Colombia or Comoros or Comores or Comoro or Congo or Costa Rica or Cote d'Ivoire or Cuba6).mp.
3. (Djibouti or Dominica or Dominican Republic or Ecuador or Egypt or El Salvador or Eritrea or Ethiopia or Fiji or Gabon or Gambia or Gaza or Georgia Republic or Georgian or Ghana or Grenada or Grenadines or Guatemala or Guinea or Guinea Bisau or Guyana).mp.
4. (Haiti or Herzegovina or Hercegovina or Honduras or India or Indonesia or Iran or Iraq or Jamaica or Jordan or Kazakhstan or Kenya or Kiribati or Korea or Kosovo or Kyrgyz or Kirghizia or Kirghiz or Kirgizstan or Kyrgyzstan).mp.
5. (Lao PDR or Laos or Lebanon or Lesotho or Liberia or Libya or Macedonia or Madagascar or Malawi or Malay or Malaya or Malaysia or Maldives or Mali or Marshall Islands or Mauritania or Mauritius or Mexico or Micronesia or Moldova or Mongolia or Montenegro or Morocco or Mozambique or Myanmar).mp.
6. (Namibia or Nepal or Nicaragua or Niger or Nigeria or Pakistan or Palau or Panama or Papua New Guinea or Paraguay or Peru or Philippines or Phillippines or Philipines or Phillipines or Principe or Romania or Rwanda or Ruanda or Samoa or Sao Tome or Senegal or Serbia or Sierra Leone or Solomon Islands or Somalia or South Africa or South Sudan or Sri Lanka or St Lucia or St Vincent or Sudan or Suriham or Suriname or Swaziland or Syria or Syrian Arab Republic).mp.
7. (Tajikistan or Tadzhikistan or Tadjikistan or Tadzhik or Tanzania or Thailand or Timor or Togo or Tonga or Tunisia or Turkey or Turkmen or Turkmenistan or Tuvalu or Uganda or Ukraine or Uzbek or Uzbekistan or Vanuatu or Vietnam or West Bank or Yemen or Zambia or Zimbabwe).mp.
8. 1 or 2 or 3 or 4 or 5 or 6 or 7
9. exp Pregnancy/
10. exp Pregnancy Complications/
11. exp Pregnancy Trimesters/
12. antenatal.mp.
13. perinatal.mp.
14. pregnan*.mp.
15. prenatal.mp.
16. trimester.mp.
17. 9 or 10 or 11 or 12 or 13 or 14 or 15 or 16
18. exp Anxiety/
19. exp Anxiety Disorders/
20. ("anxiety scale" or "anxiety inventory").mp.

21. anxiety.mp.
22. anxious.mp.
23. concerns.mp.
24. fears.mp.
25. worry.mp.
26. worries.mp.
27. ((antenatal or perinatal or pregnan* or prenatal or trimester) adj4 (anxiety or anxious or concerns or fears or worry or worries)).mp.
28. 18 or 19 or 20 or 21 or 22 or 23 or 24 or 25 or 26 or 27
29. exp Stress, Psychological/
30. stress.mp.
31. distress.mp.
32. 29 or 30 or 31
33. 28 or 32
34. 8 and 17 and 33
